# Tackling the respiratory abnormalities of Rett syndrome

**Published:** 2014-09

Rett syndrome (RTT) is a neurodevelopmental disorder caused by loss-of-function mutations in *MECP2*, which encodes the transcriptional regulator methyl-CpG-binding protein 2. Its core symptoms include problems with breathing control, which are most severe during wakefulness and are further exacerbated by behavioural arousal. Reduced levels of brain-derived neurotrophic factor (BDNF) are thought to be involved in the aetiology of respiratory dysfunction in RTT. Now, Katz et al. report that acute administration of LM22A-4, a partial BDNF mimetic, completely reverses spontaneous apnoeas (atypical breathing pauses) and respiratory dysregulation during behavioural arousal in *Mecp2* mutant mice, an RTT model. Moreover, patch-clamp recordings in *Mecp2*-null brainstem slices indicate that LM22A-4 decreases synaptic hyperexcitability within specific cell groups in the brainstem’s respiratory network. These findings support the hypothesis that reduced BNDF signalling and respiratory dysfunction in RTT are linked and demonstrate the feasibility of improving neurological function in RTT by redressing imbalances in synaptic excitability. **Page 1047**

